# Diagnostic Value of Leukocyte Count, C-Reactive Protein, and Procalcitonin in Pediatric Liver Transplant Patients During the First Week Postoperative: A Longitudinal Study from a Tertiary Center and a New Diagnostic Method for Predicting Systemic Infection

**DOI:** 10.3390/children12091201

**Published:** 2025-09-09

**Authors:** Zerrin Onal, Elif Turkmen, Demet Demirkol, Ugur Can Leblebici, Ibrahim Kandemir, Tugce Goksu Yilmaz, Selda Hancerli Torun, Feza Ekiz, Ilgin Ozden, Ozlem Durmaz

**Affiliations:** 1 Department of Pediatric Gastroenterology and Hepatology, Istanbul Faculty of Medicine, Istanbul University, Istanbul 34126, Turkey; elif.sag@istanbul.edu.tr (E.T.); canleblebici@istanbul.edu.tr (U.C.L.); tugce.yilmaz@istanbul.edu.tr (T.G.Y.); odsuoglu@istanbul.edu.tr (O.D.); 2 Pediatric Intensive Care Unit, Istanbul Faculty of Medicine, Istanbul University, Istanbul 34126, Turkey; ddemirkol@istanbul.edu.tr; 3 Department of Pediatrics, Istanbul Health and Technology University, Istanbul 34275, Turkey; ibrahim.kandemir@istun.edu.tr; 4 Pediatric Infectious Disease Unit, Department of Pediatrics, Istanbul Faculty of Medicine, Istanbul University, Istanbul 34126, Turkey; selda.hancerli@istanbul.edu.tr; 5 Departments of General Surgery, Istanbul Faculty of Medicine, Istanbul University, Istanbul 34126, Turkey; feza.ekiz@istanbul.edu.tr (F.E.); ilginozden@istanbul.edu.tr (I.O.)

**Keywords:** infection, pediatric liver transplantation, procalcitonin

## Abstract

**Highlights:**

Infectious complications are the most feared and serious problems in children undergoing liver transplantation caused by immunosuppression. Inflammatory markers rise early after the transplant due to both infection and surgical stress, which can create diagnostic challenges. Therefore, timely diagnosis of infections and prompt treatment are crucial. However, ruling out non-infectious causes is also important to prevent unnecessary antibiotic use and colonization by resistant microorganisms.

**What are the main findings?**

- Procalcitonin levels were significantly elevated on the 1st, 4th, and 7th days, while WBC levels were only elevated on the 7th day in patients with culture-positive bacterial infections.
- First day body temperature of ≥37.1 °C and a PCT of ≥5.1 ng/dL had 72.2% sensitivity and 84.3% specificity for bacterial growth, while a body temperature of ≥37.1 °C and a PCT of ≥9.2 ng/dL showed 73% sensitivity and 95% specificity for SIRS.

**What is the implication of the main finding?**

- The diagnosis of infection in pediatric LT patients increases with higher PCT levels and body temperature on the first day. We also saw the same pattern in patients with SIRS. Additionally, while PCT elevation was a significant indicator of bacterial growth, CRP and WBC values were not reliable for detecting infection.
- Elevated PCT levels and a body temperature of ≥37.1 °C on the first day may indicate bacterial growth and SIRS.

**Abstract:**

**Background/Objectives:** Infection is a major complication during the early postoperative period following pediatric liver transplantation (LT). Prompt diagnosis and treatment are essential to prevent death. We aimed to assess the diagnostic value of procalcitonin (PCT), white blood cell count (WBC), and C-reactive protein (CRP) levels for detecting bacterial infection and SIRS within the first week after LT. **Methods:** Demographic data, vital signs, laboratory results (PCT, CRP, WBC), and culture findings on the 1st, 4th, and 7th days between January 2010 and 2024 were collected retrospectively. **Results:** The study included 88 patients. Seventy-two percent had cirrhotic liver disease. SIRS was present in 17% (n = 15), and bacterial growth was detected in 20% (n = 18). Bacterial growth was found in 80% of patients with SIRS (n = 12/15) and in 8% of SIRS-negative patients (n = 6/73). PCT levels were significantly elevated on days 1, 4, and 7, and CRP levels on days 4 and 7 in patients with SIRS. PCT elevation was significant on days 1, 4, and 7, while WBC increase was noted on day 7 in patients with bacterial growth. Body temperature ≥ 37.1 °C and PCT ≥ 5.1 ng/dL on the first day together showed a sensitivity of 72.2% and specificity of 84.3% for bacterial growth. Similarly, body temperature ≥ 37.1 °C and PCT ≥ 9.2 ng/dL on the first day yielded 73% sensitivity and 95% specificity for diagnosing SIRS. Cirrhotic patients exhibited limited or no WBC response to bacterial growth. **Conclusions:** PCT, CRP, and WBC alone are insufficient for diagnosing infection. Combined assessment of body temperature changes and PCT elevation may serve as more accurate indicators. Nonetheless, close monitoring of culture results and clinical signs, with serial physical exams, can aid timely infection management or prevent unnecessary antibiotic adjustments.

## 1. Introduction

Infectious complications are among the most serious issues faced during the early postoperative period in pediatric liver transplant recipients and can elevate the risk of morbidity and mortality. These patients are vulnerable to infections related to major surgery, immunosuppressive medications, multiple catheter placements, transfusions, and impaired nutritional status during the peri-transplantation phase. They require close monitoring for both organ rejection and infectious issues, especially after surgery. In the early stages following liver transplantation (LT), infections often do not produce typical signs and symptoms. As a result, prompt and precise diagnosis, along with suitable antimicrobial therapy, is crucial to prevent sepsis and death. Furthermore, careful management of immunosuppression levels during this period is essential, since high levels can worsen infections, whereas low doses may trigger organ rejection [1].

Immunosuppressive drugs impact the humoral and cellular immune systems and can lead to serious complications after a transplant. Systemic infection is one of the most common and potentially life-threatening complications. Therefore, early detection and treatment of systemic infections within the first week after surgery are essential. Despite many valuable studies, there are currently no standardized diagnostic protocols for identifying infections in pediatric patients who have recently had a liver transplant (LT). Procalcitonin (PCT), white blood count (WBC), and C-reactive protein (CRP) are the most commonly used biomarkers for detecting infection. However, since these markers can also increase in response to stress from trauma, burns, or surgery, diagnostic challenges may occur [1,2].

Procalcitonin is the precursor of the hormone calcitonin, produced by C cells in the thyroid gland of healthy individuals. In cases of inflammation, it is produced by neuroendocrine cells in non-thyroid tissues such as the lungs, liver, and kidneys [3]. It is usually not present in significant amounts in the bloodstream of healthy people. PCT levels are unaffected by viral infections or acute cellular rejection. Therefore, it is a valuable biomarker for bacterial infections, showing greater specificity and sensitivity than other inflammatory markers like CRP and WBC, and it helps distinguish bacterial infections from other complications [3,4,5,6]. After hepatobiliary surgery, serum PCT levels may increase due to hepatic storage and the systemic inflammatory response caused by surgical trauma, ischemia–reperfusion injury, and potential bacterial translocation. This temporary rise typically peaks within 24–48 h and then declines rapidly if no complications occur. However, persistently high or secondary rises in PCT levels may indicate postoperative problems such as surgical site infections, anastomotic leaks, or sepsis [2,4]. C-reactive protein is an acute-phase reactant and biomarker, but it is less specific and sensitive than PCT. It is used to detect significant inflammation or infection in children. During the early postoperative period, CRP levels may rise due to systemic inflammatory response syndrome (SIRS). Nonetheless, impaired synthesis from liver injury can lead to falsely low CRP levels, potentially hiding the presence of infection even when it exists [2,7].

Systemic inflammatory response syndrome (SIRS) is an inflammatory reaction to stress within the body rather than caused by infectious or non-infectious factors, such as trauma, burns, and surgery. Although the clinical signs are similar, the main difference between SIRS and sepsis is that no microorganisms are detected in cultures. Since LT is a major surgical procedure, SIRS often occurs after LT [8].

Procalcitonin, WBC, and CRP levels have not been compared in children during the postoperative period after LT.

In this retrospective study, our goal is to assess the diagnostic value of PCT, WBC, and CRP elevations in detecting bacterial infections and differentiating between infection and SIRS in the early postoperative period after LT.

## 2. Materials and Methods

We included patients aged 1–18 years who were followed before and immediately after LT at the Istanbul University Pediatric Liver Transplantation Unit from January 2010 to January 2024. Only patients with data available during the first week after LT were part of the study. Demographic features, vital signs, laboratory results (PCT, CRP, WBC), and bacterial growth in cultures on the 1st, 4th, and 7th days were retrospectively collected from hospital records. All patients received prophylactic antibiotics preoperatively and postoperatively as part of the routine clinical protocol.

Serum PCT level was measured using the Elecsys BRAHMS PCT assay (Roche Diagnostics, Mannheim, Germany) on a cobas e 411 analyzers. Serum PCT levels (<0.1 ng/mL; low risk, 0.5–2 ng/mL; moderate risk, >2 ng/mL; high risk), CRP (normal range 0.0–5 mg/dL), and WBC counts were compared in patients with and without SIRS and bacterial growth in culture. Standard age-related cutoffs were used to determine elevated WBC [9]

Infection screening was performed on all patients by analyzing ascitic fluid, urine, and blood culture samples. SIRS was defined according to the International Pediatric Sepsis Consensus Conference criteria. The presence of two or more of the following four criteria indicated SIRS: (i) abnormal core temperature (greater than 38.5 °C or less than 36 °C), (ii) tachycardia (mean heart rate more than two standard deviations above normal for age, without external stimulus, chronic medication, or painful stimuli), (iii) tachypnea (mean respiratory rate more than two standard deviations above normal for age) or mechanical ventilation, and (iv) abnormal white blood cell count (either elevated or decreased) for age [10].

The study was conducted in accordance with the principles of the Helsinki Declaration, and approval was obtained from the ethics committee of Istanbul Faculty of Medicine (Ethics Committee no: 2024/2000).

All calculations in our study were performed using the Jamovi statistical software version 2.3.18 with the “GAMLj” extension. Normally distributed continuous variables were expressed as mean ± standard deviation (SD), non-normally distributed continuous variables as median (range). Categorical variables are used as percentages (%). The comparison of quantitative data between groups was conducted using the Student *t*-test for normally distributed variables and the Mann–Whitney test for non-normally distributed variables. Qualitative data were compared using the Chi-square test. We employed the generalized linear model in multivariate analysis and used the likelihood ratio test for significance testing. All factors were entered as “dummies” into the multivariate models, and confounding factors were assessed through the “backward elimination” method with a *p*-value cutoff of <0.2. The estimated marginal means table with 95% confidence intervals (95% CI) was presented, keeping other factors constant at their mean values. Repeated-measures ANOVA was performed to evaluate changes, using transformed data to meet the necessary assumptions. For clarity, figures display raw data. Statistical significance was set at *p* ≤ 0.05.

## 3. Results

A total of 88 patients [53% female, median age 49 months (range 18–95 months)] were included in the study. Seventy-two percent of the patients were cirrhotic (n = 63), and 28% had non-cirrhotic liver disease (n = 28), with 15% having metabolic disease and 13% other causes. Fifty-six (90%) of the cirrhotic patients had cholestatic liver disease, 12 (19%) experienced portal hypertension complications, and 9 (14%) had a poor quality of life, including growth retardation and itching. Indications for liver transplantation included decompensated chronic liver disease (n = 59, 67%), acute liver failure (n = 12, 14%), metabolic disease (n = 13, 15%), and persistent portal hypertension complications despite conservative treatment, as well as poor quality of life due to secondary issues like itching, fat-soluble vitamin deficiency, and growth retardation (n = 4, 4%). Living related donors were used in 94% of cases, while only 6% of children received a cadaveric organ.

Patients were classified based on the presence or absence of SIRS and bacterial growth in cultures. SIRS was present in 17% (n = 15) of patients, while bacterial growth was observed in 20% (n = 18). Among patients with SIRS (+), 80% (12/15) had bacterial growth, compared to only 8% (6/73) of those without SIRS (−). Hemocultures and/or abdominal fluid samples detected Klebsiella, Enterococcus, *E. coli*, and methicillin-resistant coagulase-negative Staphylococcus.

Body temperature on the first day was significantly higher in patients with SIRS and bacterial growth (*p* < 0.001, *p* < 0.001). There was no significant difference on the 4th and 7th days.

When patients were assessed for SIRS, PCT levels were significantly higher on the 1st, 4th, and 7th days (*p* < 0.01), while CRP was notably elevated on the 4th and 7th days (*p* < 0.05) in patients with SIRS (+). No significant difference was observed between the groups for WBC on any day.

When analyzing bacterial growth in patients, PCT levels were significantly elevated on days 1, 4, and 7. In contrast, WBC levels increased only on day 7 in patients with culture-positive bacterial infections. There was no statistical difference between the groups regarding CRP on any day. The clinical and laboratory findings of the patients are shown in Table 1.

**Figure 1:**
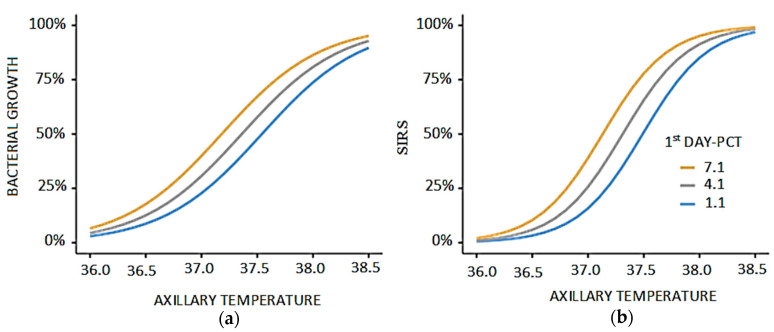
The correlation of SIRS with axillary temperature and PCT levels on the first day. (**a**) Relationship between axillary temperature and bacterial growth stratified by first-day PCT levels. Bacterial proliferation shows a gradual increase with rising axillary temperature, reaching a sharper elevation beyond 37.5 °C. The effect is more pronounced in patients with higher PCT levels (7.1 and 4.1 ng/mL), indicating enhanced bacterial activity compared to those with lower PCT (1.1 ng/mL). (**b**) Relationship between axillary temperature and SIRS stratified by first-day PCT levels. The probability of SIRS rises steadily with increasing axillary temperature, with a more marked elevation beyond 37.5 °C. Higher PCT levels (7.1 and 4.1 ng/mL) are associated with an earlier and stronger increase in SIRS compared to lower PCT values (1.1 ng/mL).

We included factors such as age, gender, cirrhosis, metabolic diseases, cholestasis, and the use of immunosuppressive drugs (two or three medications) as dummy variables in a multivariate model to assess potential confounders. Body temperature and PCT level showed independent associations with SIRS and bacterial growth on day one. The odds of SIRS increased by 1.22 times (95% CI: 1.02–1.54) for each one ng/mL increase in PCT and by 30.4 times (95% CI: 6.2–292.3) for each 1 °C increase in body temperature (generalized linear model, R2: 0.448). The odds of bacterial growth rose by 1.14 times (95% CI: 1.0–1.4) for each one ng/mL increase in PCT and by 9.5 times (95% CI: 2.8–45.5) for each 1 °C increase in body temperature (generalized linear model, R2: 0.264). The figure and the estimated marginal means tables are included in Figure 1 and Table 2.

**Figure 2:**
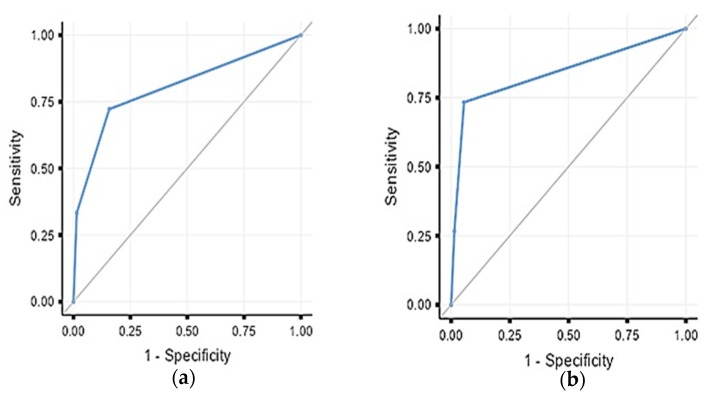
(**a**) ROC analysis for bacterial growth prediction with fever exceeding 37.0 °C or PCT exceeding 5.1 on the first postoperative day. (**b**) ROC analysis for SIRS prediction with fever exceeding 37.0 °C or PCT exceeding 9.2 on the first postoperative day.

First-day body temperature and PCT levels were included in the ROC analysis to determine the best model for early prediction (Figure 2). We found that a first-day body temperature of ≥37.1 °C and PCT of ≥5.1 ng/dL had 72.2% sensitivity and 84.3% specificity for bacterial growth (AUC: 0.80) (Table 3). Conversely, the diagnostic performance was slightly better for SIRS, with a first-day body temperature of ≥37.1 °C and PCT of ≥9.2 ng/dL, which showed 73% sensitivity and 95% specificity for SIRS (AUC: 0.84) (Table 4).

### ANOVA Analysis

**Figure 3:**
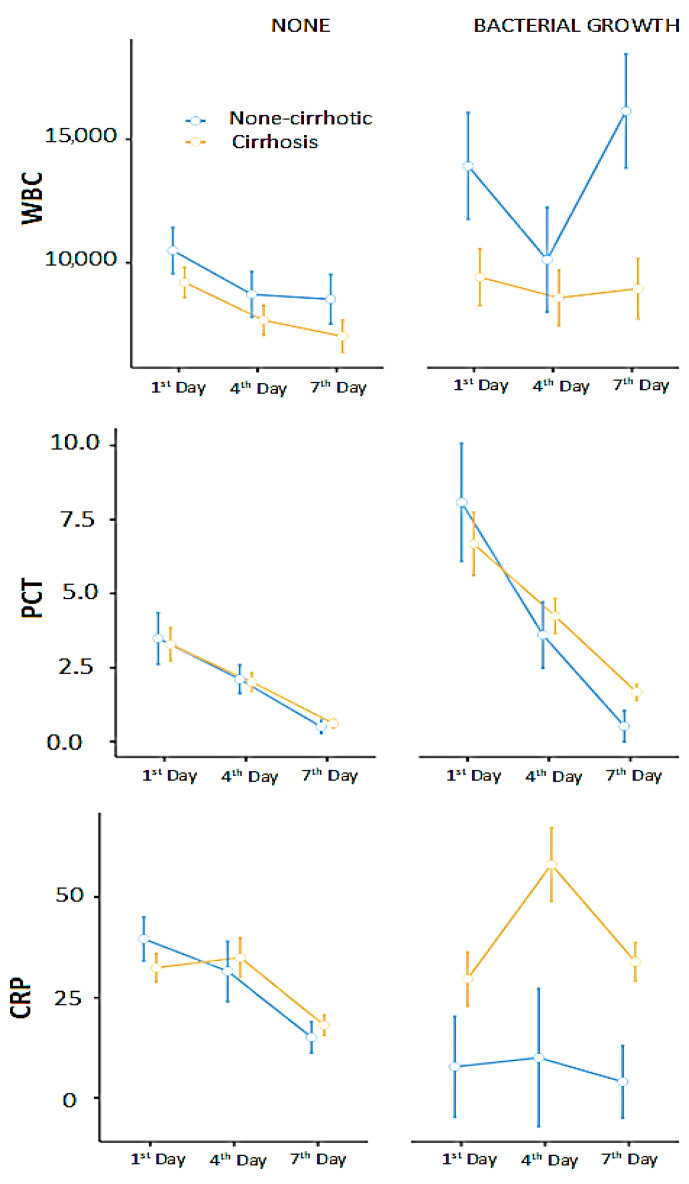
WBC, CRP, and PCT responses to bacterial infections in cirrhotic and non-cirrhotic. The figure is prepared with raw (non-transformed) data for better presentation. The bars represent standard errors.

We built repeated measures ANOVA models to evaluate changes in WBC, PCT, and CRP in relation to bacterial growth and cirrhosis. Cirrhosis (*p* = 0.028) and bacterial growth (*p* = 0.035) significantly affected WBC levels; however, the interaction between bacterial growth and cirrhosis (i.e., bacterial growth in patients with cirrhosis) was not statistically significant (*p* = 0.190). PCT was only significantly influenced by bacterial growth (*p* = 0.001). CRP was significantly affected by cirrhosis (*p* = 0.007) and the bacterial growth*cirrhosis interaction (*p* = 0.014) (Figure 3).

Pairwise comparisons showed significant differences in WBC levels between the 1st and 4th days (*p* = 0.001), between patients with and without cirrhosis (*p* = 0.017), in cases of bacterial growth (*p* = 0.03), and between non-cirrhotic patients with bacterial growth and cirrhotic patients without bacterial growth (*p* = 0.03).

PCT levels showed significant differences between the 1st and 7th days (*p* < 0.001), between the 4th and 7th days (*p* = 0.004), and in the presence of bacterial growth (*p* = 0.033).

Similarly, CRP levels differed significantly between the 1st and 7th days (*p* = 0.007), between the 4th and 7th days (*p* < 0.001), between cirrhotic and non-cirrhotic patients (*p* = 0.009), and between non-cirrhotic patients with bacterial growth and cirrhotic patients with bacterial growth (*p* = 0.02).

The graphical analysis suggested that cirrhotic patients might have an insufficient immune response to infection (bacterial growth). Therefore, we examined changes in WBC, PCT, and CRP levels in these patients with bacterial growth. Bayesian analysis showed moderate evidence supporting the null hypothesis (H_0_) for WBC (BF_10_ = 0.209), while PCT (BF_10_ = 1.13) and CRP (BF_10_ = 2.14) provided anecdotal evidence favoring the alternative hypothesis (H_1_), indicating a possible association. Cirrhotic patients showed significant changes in PCT and CRP during bacterial growth (based on anecdotal evidence); however, WBC levels did not respond to bacterial growth (more evidence against).

## 4. Discussion

This study examined the diagnostic value of PCT, CRP, and WBC during the early postoperative period in pediatric liver transplantation (LT). Our results indicated that diagnosing infection in pediatric LT patients is more probable when PCT levels and body temperature are elevated on the first day. We also observed a similar pattern in patients with SIRS. Furthermore, although an increase in PCT was a significant marker for bacterial growth, CRP and WBC levels were not definitive indicators of infection.

We recognize that these biomarkers naturally fluctuate during post-operative recovery and infection treatment, which can lower the interpretive value of isolated comparisons. During the first week, PCT, CRP, and WBC levels may vary due to the recovery process and are not reliable indicators of bacterial infections. However, clinical symptoms such as fever are more important. We have shown that combining changes in body temperature and PCT increases diagnostic accuracy during this critical period. In a study on bacteremia after LT, a PCT cut-off value of 2.0 ng/mL demonstrated a positive PPV of 83.3%. The authors suggested that patients with PCT levels above 2.0 ng/mL are more likely to have bacteremia, especially after the seventh day post-transplant [1]. A recent study from Egypt found that measuring combined PCT and WBC levels, with cutoffs of <9 ng/mL and <17,300/mm^3^, respectively, helped exclude infections in 83.7% of patients on day one after LT [11]. In a systematic review, Jerome et al. reported pooled sensitivity (70%) and specificity (77%) with a diagnostic odds ratio of 15.8 for PCT. However, it was concluded that high heterogeneity existed due to differences in sampling timing (post-operative days 1–8) and the wide range of cut-off values (0.48 to 42.8 ng/mL) across studies [12]. In our study, the most accurate predictive values for infection were obtained by combining elevated procalcitonin levels with changes in body temperature. Nevertheless, these tests produced similar results in patients with SIRS. Therefore, we recommend close monitoring of cultures, clinical findings, and serial physical exams to prevent unnecessary antibiotic use when infection is suspected.

In our series, 20% of LT recipients experienced postoperative bacterial growth. In an adult study, Saner et al. reported bacterial growth in 32.7% (18/55) of living donor LT recipients and 20.8% (36/173) of cadaveric LT recipients [13]. Kim et al. found that 24% of liver donor LT recipients developed bacteremia in their cohort [14]. In a pediatric study, it was observed that 41% (17/48) of patients developed bacterial infections within the first week after pediatric LT. The most common pathogen was Enterococcus faecium [15]. Reported infection rates may vary depending on differences in the preoperative condition of recipients, such as MELD/PELD scores, nutritional status, and perioperative factors. Proper monitoring and early intervention strategies are essential for reducing the risk of infection and facilitating timely diagnosis.

In addition to bacterial infections, invasive fungal infections (IFIs) are a major cause of morbidity and mortality after liver transplantation. Candidiasis (60–80%) and aspergillosis (1–8%) are the most common mycoses, with associated mortality rates of 30% to 50% and 65% to 90%, respectively.

Large cohort studies on solid organ transplantation recipients showed a 1-year post-transplant IFD rate of 4–8% [16,17,18]. The incidence of early IFIs in pediatric liver transplant recipients was reported as 4.7% and 2%, respectively. Broad-spectrum antibiotic therapy, parenteral nutrition, prolonged neutropenia, ICU stay, pre-LT colonization, renal replacement therapy, cytomegalovirus (CMV) infection, re-interventions and choledochojejunostomy, surgical time greater than 11 h are noted risk factors for post-LT IFIs. Antifungal prophylaxis is recommended during the first month post–transplantation in high–risk patients, particularly those who received a living donor transplantation and were treated with antibiotics [19]. Phichaphop et al. found IFI to be 31% (n = 42), mortality 5% (n = 2) in the 136 patients. The median time from LT to IFI occurrence was 7.5 days (IQR: 4, 16). Among 41 fungal isolates, *Candida albicans* was the most common fungal infection (56%) [20]. In our series, we did not determine any fungal infections. We believe that IFI was not observed because antifungal prophylaxis was applied during and after the operation.

In children with liver disease, the presence of SIRS is often linked to infection. In a study, 21.9% of children with liver disease met the criteria for SIRS, and 86% of them had an infection [21]. In our study, 17% of patients were SIRS-positive, with 80% of these showing bacterial growth. Only three children out of 15 had SIRS without infection. SIRS and infection are very difficult to distinguish because they share similar clinical features. During this period, monitoring culture results is important for diagnosing infection and preventing unnecessary prolonged antibiotic use by starting the correct treatment at the right time. Notably, in our study, PCT levels were higher in patients with SIRS despite the absence of infection. This highlights the importance of avoiding unnecessary antibiotics when there is no infection. Unnecessary antibiotic use after transplantation negatively affects the patient’s microbiota and increases the risk of colonization with multidrug-resistant bacteria. Therefore, early diagnosis of infection and timely initiation of targeted treatment are essential.

The retrospective design and analysis of data from pediatric liver recipients within the first seven days after LT are limitations of our study. The diagnostic performance of different biomarkers also needs evaluation after the seventh day. We recognize and account for the overlap between the SIRS and bacterial infection groups in our analysis. It is well understood that patients meeting the clinical criteria for SIRS and testing positive for bacterial infections via culture generally present fewer diagnostic challenges. In such cases, empirical antibiotics are followed by culture-guided definitive therapy, which follows a standard clinical pathway. Future studies with larger sample sizes could greatly help refine diagnostic and treatment strategies, potentially leading to better patient outcomes in critical care.

In conclusion, PCT, CRP, and WBC are not specific for diagnosing infection in the first week after pediatric LT and should be evaluated alongside clinical symptoms, especially changes in body temperature. Among the considered biomarkers that indicate infectious complications, only PCT may serve as a reliable indicator compared to CRP and WBC on days 1, 4, and 7 for culture-positive bacterial infections. It is also recommended to monitor PCT over time rather than relying on a single measurement for decision-making. Additionally, it is essential to recognize that SIRS can cause significant PCT elevation and body temperature fluctuations, even in the absence of a culture-positive infection. Close monitoring of cultures, clinical signs, and serial physical exams remains crucial for timely and appropriate infection management and to prevent unnecessary antibiotic use. Further studies involving larger patient groups are planned to determine the significant thresholds for relevant biomarkers to improve diagnostic accuracy for bacterial infections.

## Figures and Tables

**Table 1:** Clinical and laboratory findings of the patients on the 1st, 4th, and 7th day.

		SIRS			Bacterial Growth		
		SIRS (−) n:73	SIRS (+) n:15	*p*	Growth (−) n:70	Growth (+) n:18	*p*
**Age, month, median (min–max)**		55 (19–111)	23 (10–62)	0.054	53 (19–108)	34 (15–81)	0.196
**First day fever, °C (mean ± SD)**		36.5 ± 0.3	37.3 ± 0.7	**<0.001**	36.5 ± 0.3	37.1 ± 0.7	**<0.001**
**1st day** **median (min–max)**	**WBC (×10^3^/µL)**	9750 (6540–12,300)	9640 (7810–14,000)	0.374	9630 (6758–12,413)	9745 (6573–13,036)	0.481
	**PCT (ng/mL)**	2.7 (1.6–4.2)	5.6 (2.5–10.8)	**0.008**	2.8 (1.6–4.1)	5.2 (2.4–9.4)	**0.017**
	**CRP (mg/dL)**	25 (15–49)	16 (7–37)	0.147	26 (15–49)	18 (8–33)	0.063
**4th day** **median (min–max)**	**WBC (×10^3^/µL)**	7300 (5250–9900)	8740 (4925–14,610)	0.191	7270 (5268–10,125)	8420 (4838–10,508)	0.406
	**PCT (ng/mL)**	1.6 (0.8–2.3)	2.9 (1.7–7.7)	**0.006**	1.7 (0.8–2.3)	2.7 (1.4–6.3)	**0.021**
	**CRP (mg/dL)**	23 (15–43)	41 (23–83)	**0.046**	24 (15–45)	34 (15–63)	0.535
**7th day** **median (min–max)**	**WBC (×10^3^/µL)**	6760 (4320–9200)	8470 (5920–13,325)	0.149	6555 (4148–8898)	9485 (6770–15,055)	**0.018**
	**PCT (ng/mL)**	0.3 (0.2–0.5)	1.3 (1.0–2.7)	**<0.001**	0.4 (0.2–0.6)	0.9 (0.5–1.5)	**0.002**
	**CRP (mg/dL)**	11 (8–18)	29 (14–56)	**0.004**	11 (8–21)	15 (6–42)	0.468

**CRP**: C-reactive protein, **PCT**: procalcitonin, **SIRS**: systemic inflammatory response syndrome, **WBC**: white blood cell.

**Table 2:** Estimated marginal means for SIRS and bacterial growth, along with body temperature and PCT levels on the first day.

		SIRS	Bacterial Growth
**1st day—PCT (ng/mL)**	1.1	4.5% (1.2–15.1)	10.6% (4.6–22.5)
	2.1	5.5% (1.8–15.9)	12.0% (5.8–23.2)
	3.1	6.6% (24.1–17.0)	13.5% (7.1–24.2)
	4.1	3.7% (3.2–18.7)	15.1% (8.3–25.9)
	5.1	9.7% (4.1–21.1)	17% (9.5–28.5)
	6.1	11.6% (4.8–24.7)	19.0% (10.4–31.9)
	7.1	13.9% (5.8–29.7)	21.1% (11.1–36.4)
	8.1	16.4% (6.4–36.0)	23.4% (11.6–41.7)
**1st day—BT (Celsius)**	36.1	1.6% (0.3–7.6)	5.5% (1.9–14.8)
	36.3	3.1% (0.8–10.7)	8.4% (3.6–18.2)
	36.5	5.9% (2.1–15.4)	12.6% (6.5–22.9)
	36.7	11.1% (4.9–23.2)	18.4% (10.6–30.0)
	36.9	19.7% (9.5–36.5)	26.1% (15.3–40.8)
	37.1	29.5% (14.2–51.5)	33.4% (19.0–51.8)
	37.3	49.0% (22.6–76.0)	46.5% (24.6–69.8)
	37.5	67.8% (31.4–90.6)	59.3% (29.9–83.3)

**BT**: body temperature, **PCT**: procalcitonin, **SIRS**: systemic inflammatory response syndrome.

**Table 3:** Sensitivity, specificity, and likelihood ratios of first-day body temperature and PCT as indicators of bacterial growth during the postoperative LT period.

	Cut Point	Sensitivity (%)	Specificity (%)	PPV (%)	NPV (%)	AUC
**1st day—PCT**	4.4	55.56	81.43	43.48	87.69	0.684
	4.5	55.56	82.86	45.45	87.88	0.684
	**5.1**	**55.56**	**85.71**	**50**	**88.24**	0.684
**1st day—BT**	36.6	83.33	65.71	38.46	93.88	0.796
	36.9	61.11	87.14	55	89.71	0.796
	**37.1**	**50**	**97.14**	**81.82**	**88.31**	0.796
**1st day BT + PCT**	**1**	**72.22**	**84.29**	**54.17**	**92.19**	0.804

**AUC**: area under the curve, **BT**: body temperature **PCT**: procalcitonin, **PPV**: positive predictive value, **NPV**: negative predictive value.

**Table 4:** Sensitivity, specificity, and likelihood ratio of first-day body temperature and PCT as an indicator of SIRS in the postoperative LT period.

	Cut Point	Sensitivity (%)	Specificity (%)	PPV (%)	NPV (%)	AUC
**1st day—PCT**	5.1	53%	84%	40%	90%	0.718
	5.2	53%	85%	42%	90%	0.718
	5.6 **9.2**	53% **40%**	86% **96%**	44% **67%**	90% **89%**	0.718 **0.718**
**1st day—BT**	36.9	73%	88%	55%	94%	0.868
	37	671%	95%	71%	93%	0.868
	**37.1**	**60%**	**97%**	**81%**	**92%**	0.868
**1st day BT + PCT**	**1**	**73%**	**95%**	**73%**	**95%**	**0.842**

AUC: area under the curve, BT: body temperature, **PCT**: procalcitonin, **PPV**: positive predictive value, **NPV**: negative predictive value.

## Data Availability

The data that support the findings of this study are available from the corresponding author upon reasonable request.

## Abbreviations

AUC: area under the curve, CRP: C-reactive protein (CRP), LT: liver transplantation, NPV: negative predictive value, PPV: positive predictive value, PCT: procalcitonin, SIRS: systemic inflammatory response syndrome, WBC: white blood count.
